# Selection of *Ceratitis capitata* (Diptera: Tephritidae) Specific Recombinant Monoclonal Phage Display Antibodies for Prey Detection Analysis

**DOI:** 10.1371/journal.pone.0051440

**Published:** 2012-12-14

**Authors:** César Monzó, Alberto Urbaneja, Miguel Ximénez-Embún, Julia García-Fernández, José Luis García, Pedro Castañera

**Affiliations:** 1 Department of Environmental Biology, Centro de Investigaciones Biológicas, CSIC, Madrid, Spain; 2 Unidad Asociada de Entomología IVIA-CIB, CSIC, Centro de Protección Vegetal y Biotecnología, Instituto Valenciano de Investigaciones Agrarias, Valencia, Spain; The Scripps Research Institute, United States of America

## Abstract

Several recombinant antibodies against the Mediterranean fruit fly, *Ceratitis capitata* (Wiedemann) (Diptera: Tephritidae), one of the most important pests in agriculture worldwide, were selected for the first time from a commercial phage display library of human scFv antibodies. The specificity and sensitivity of the selected recombinant antibodies were compared with that of a rabbit polyclonal serum raised in parallel using a wide range of arthropod species as controls. The selected recombinant monoclonal antibodies had a similar or greater specificity when compared with classical monoclonal antibodies. The selected recombinant antibodies were successfully used to detect the target antigen in the gut of predators and the scFv antibodies were sequenced and compared. These results demonstrate the potential for recombinant scFv antibodies to be used as an alternative to the classical monoclonal antibodies or even molecular probes in the post-mortem analysis studies of generalist predators.

## Introduction

The trophic relationships between predators and their prey can be studied through a number of techniques including post-mortem gut content analyses. Methodologies for these studies comprise the use of protein electrophoresis, prey-specific protein antibodies or DNA analysis. In post-mortem studies the most accurate information is obtained using either serological methods or molecular markers [1].

Polyclonal antibodies have been used in predation post-mortem studies for more than 60 years [2]–[5]. These antibodies can be obtained in a relatively short period of time and are inexpensive to produce but do not show high specificity and the serum obtained is unique and limited [1], [6]. Due to the lack of specificity, these sera need to be tested against a wide range of alternative preys in order to establish the lower threshold for positive detection when used to study predatory-prey systems [7].

Monoclonal antibodies originate from a single β-lymphocyte cell line and are specific for one type of epitope [8]. Köhler and Milstein [9] developed the hybridoma technique that allows for limitless production of homogeneous monoclonal antibody sera. Monoclonal antibodies are considered one of the most useful tools in post-mortem gut content analysis as a result of their high sensitivity and specificity [10], [11]. The hybridoma technique has allowed for the development of prey specific antibodies to any taxonomical level or even developmental stage. In fact, monoclonal sera have been used to determine predation across different developmental stages of the same arthropod [12], [13]. The use of monoclonal antibodies to detect epitopes with low lability or epitopes that appear once digestion has begun, has allowed for increased detection times [14], [15], [16]. The main disadvantages to the hybridoma system of antibody production are the need for specialized equipment and trained personnel, besides being time consuming. The high stochastic component that this technique entails does not guarantee the success of a project [17], [18].

Phage display technologies have been developed for the production of antibodies under more controlled selection conditions. During the 1990s, a technique was developed to obtain recombinant monoclonal antibodies by inserting DNA sequences that encoded the dimeric antigen binding (Fab_2_), antigen binding (Fab) and single chain variable (scFv) fragments into the genome of filamentous bacteriophage. These fragments contain the antibody regions responsible for recognition and binding of antigens. The Fab fragments are composed of one constant and one variable domain of the heavy and light chain; the Fab_2_ fragments have four variable and two constant domains from each heavy and light chain; and the scFv fragments are fusion proteins consisting of one variable domain of light and heavy chain connected with a linker peptide. Recombinant bacteriophages express the antibody fragments as fusion products on the phage coat proteins [19], [20]. Currently, this technology uses phagemids, – usually variant of M13, fd or fl. Phagemids contain *Escherichia coli* origins of replication and also phage packing signals. The *E. coli* origins of replication enable replication as double stranded DNA within the bacteria. The phage packing signals permit its packing as a single strand DNA recombinant M13, fd or fl phage in the presence of a helper phage. Helper phages provide the structural genes required for packaging, and contain a defective origin of replication. This reduction in expression permits more efficient replication and packaging of the phagemid than the helper when both are present [17]. The use of phagemids allows for the selection and enrichment of clones displaying the antibodies of interest in the capsule, when they are in the phage form, and also permits the expression of the soluble antibody fragments (usually scFv) when acting as a plasmid in *E. coli*.

Phage libraries can offer a higher diversity in antibodies (between 10^8^ and 10^10^ clones) [21] than libraries generated by an organism (between 10^5^ and 10^6^ different lymphocyte clones) [22]. This high diversity provides a greater chance of success for isolating highly specific monoclonal antibodies against a target antigen. The clone selection process for phage libraries is much quicker, more economical, and more efficient than the classic monoclonal antibodies approach. In addition, the selected recombinant antibodies can be produced indefinitely in *E. coli* or they can be sequenced, and the specificity of the antibodies can be further improved by creating new variants by direct DNA synthesis or by any other mutagenic technique [23]. Due to these characteristics, such as high specificity and limitless production, the hybridoma system can be matched by recombinant antibodies. Finally, the use of phage display technologies avoids the utilization of laboratory animals to obtain the sera, an issue that is controversial [24], [25], [26].

It has been suggested that the use of monoclonal antibodies in prey detection is the fastest and least inexpensive post-mortem technique available when a single prey species is targeted and large numbers of samples need to be evaluated [6], [27]. The use of phage display antibodies in post-mortem studies have the potential to overcome all the disadvantages inherent in obtaining animal derived monoclonal antibodies and therefore considerably reduce the costs of these kinds of surveys.

The possibility of using phage monoclonal antibodies for pest predation studies has been previously reported [28]. However, to the best of our knowledge, this is the first work selecting and sequencing recombinant antibodies from a commercial phage display library, specifics for one of the most important pests in agriculture worldwide, the Mediterranean fruit fly, *Ceratitis capitata* (Wiedemann) (Diptera: Tephritidae). The specificity and sensitivity of the selected recombinant antibodies have been compared with that of a rabbit polyclonal serum raised in parallel. The high specificity of the monoclonal antibody selected was tested with a wide range of arthropod species. Finally, feeding trials were performed with the wolf spider *Pardosa cribata* Simon (Araneae: Lycosidae), an important *C. capitata* predator in Spanish citrus orchards [29], to prove that the selected antibody clone is capable of detecting the prey antigens in the gut of the predator. The development of *C. capitata* specific recombinant antibodies on post-mortem studies of generalist predators provides important information on trophic interactions taking place in agro-ecosystems.

## Materials and Methods

### Preparation of Antigens

A total soluble protein extract of *C. capitata* was obtained by homogenization of 1 g of adult flies in a sex ratio 1∶1 in 2 ml of PBS buffer at 4°C. The soluble protein in the supernatant was assessed by Bradford Protein Assay [30]. This method was also used to prepare protein extracts of the arthropod species used for the cross-reactivity tests.

### Rabbit Polyclonal Serum

Specific polyclonal antibodies against *C. capitata* were raised in a New Zealand rabbit strain in the core facilities of CIB (CSIC, Madrid) using the previously described *C. capitata* antigen extract. Briefly, the initial subcutaneous injection consisted of 1 ml of the soluble protein extract (500 µg/ml) emulsified in 1 ml of Freund Complete Adjuvant® (SIGMA-ALDRICH Steinheim am Albuch, Germany) followed two weeks later by an intramuscular (IM) booster injection consisting of 500 µg of the antigen extract emulsified in Freud Incomplete Adjuvant® (SIGMA-ALDRICH Steinheim am Albuch, Alemania). A final IM injection was administered two weeks later and consisted of antigen extract in Freud Incomplete Adjuvant as described above. The reactivity of the serum was tested against the target antigen by Indirect ELISA prior blood collection, which was initiated two weeks after the final injection. The blood was allowed to clot for 20 h at 4°C, the serum removed following low-speed centrifugation, aliquoted and stored at −20°C until analysis. The specificity and sensitivity of different dilutions of the polyclonal serum (1/500, 1/1,000, 1/5,000 and 1/10,000) were evaluated against varying soluble protein concentrations (10, 1, 10^−1^ and 0 µg/ml) of the dipterans *C. capitata* and, a close related species, *Drosophila melanogaster* Meigen by Indirect ELISA. Each serum dilution and protein concentration combination was tested in triplicate.

### Monoclonal Recombinant Antibodies and Biopanning

Specific recombinant monoclonal antibodies against *C. capitata* were selected from two commercial recombinant phage libraries: Human Single Fold scFv I and Human Single Fold scFv J (Tomlison I+J) (Medical Research Council, HGMP Resource Centre, Cambridge, UK). Both libraries were screened in parallel to ensure selection of the maximum number of clones displaying specific binding to target antigens.

Biopanning was carried out as per manufacturer’s instructions with modifications. KM13 helper phages (2×10^11^ pfu) were used to rescue phages prior to biopanning and all procedures were performed in 96-well ELISA plate (Corning Constar®) format, with adjustments in volume and concentrations of reagents as noted below. Clone selection and enrichment was carried out using the *C. capitata* soluble protein extracts. Briefly, for each phage library, 16 wells were coated with 50 µl of coating buffer solution (10 mM NaHCO_3_ pH 9.6) containing 10 µg/ml of soluble protein. As negative controls, 8 wells were coated with 50 µl of MPBS (3% skimmed milk in PBS) per library. Plates were incubated overnight at 4°C, rinsed three times with PBS, blocked with MPBS for 2 h at room temperature and then rinsed three times with PBS. Next, 10^12^ pfu of each phagemid/ScFv library were added to assigned wells, incubated for 1 h at room temperature, and wells were vigorously rinsed 20 times with PBS-Tween20 (0.1%) followed by three additional washes with PBS. Bound antibodies were eluted in 50 µl of PBS containing 1 mg/ml of trypsin (T-1426 Type XIII, Sigma). A total of three rounds of biopanning were performed and selected clones were evaluated for specificity and sensitivity to *C. capitata* antigen.

### Sequencing, Alignment and Comparison of the V_H_ and V_κ_ Regions Encoded by the Selected Recombinant Clones

To determine the uniqueness of each selected clone, the V_H_ and V*_κ_* inserts of clones displaying the highest specificity and sensitivity were sequenced and compared. Plasmids were extracted and sent for sequencing at SECUGEN (Madrid, Spain). Chromatograms were visualized using the Staden Package [31], and sequence alignment and comparisons were done using GeneDoc [32]. Sequences encoding V_H_ and V*_κ_* regions were compared to sequences in GENBANK gene database using the BLASTN 2.2.21 application [33], [34].

### Specificity and Sensitivity Tests for the Selected Antibodies

Selected intact phages displaying unique pIII-monoclonal antibody protein were used to evaluate the specificity and sensitivity for *C. capitata* antigen. Monoclonal antibody sensitivity for selected phages was tested by indirect ELISA against varying concentrations of the *C. capitata* antigen (5 µg/ml, 2.5 µg/ml and 1 µg/ml) and different amounts of the selected phage (2E+11, 2E+10, 8E+09 and 4E+09 pfu). Each serum dilution and protein concentration combination was tested in triplicate. Additionally, antibody specificity was tested using soluble protein extracts from several closely related species (Diptera), and unrelated species (Table 1) that can be present in agroecosystems. Each soluble protein extract was tested in triplicate.

**Table 1:** Cross-reactivity test for the monoclonal recombinant antibody TJCc2.

Order	Family	Species	Absorbance (492 nm)
DIPTERA	Tephritidae	*Ceratitis capitata* (Weidenmann) [adults]	0.762±0.049 a
		[pupae]	0.592±0.067 b
	Drosophilidae	*Drosophila melanogaster* Meigen	0.027±0.006 d
	Cecidomidae	*Aphidoletes aphidimyza* Rondani	−0.002±0.003 d
	Calliphoridae	*Sarcophaga sp.*	0.183±0.020 c
	Syrphidae	*Episyrphus balteatus* (De Geer)	0.005±0.002 d
	Sciaridae	*Bradysia sp.*	0.035±0.003 d
ACARI	Phitoseiidae	*Amblyseius californicus* McGregor	−0.003±0.001 d
ARANEAE	Lycosidae	*Pardosa cribata* Simon	0.087±0.008 d
COLEOPTERA	Staphylinidae	*Philonthus quisquiliarius* (Gyllenhal)	−0.019±0.006 d
	Curculionidae	*Rhynchophorus ferrugineus* Olivier	0.010±0.002 d
HEMIPTERA	Aphidae	*Sitobion fragariae* (Walker)	−0.005±0.000 d
		*Aphis spiraecola* Patch	−0.003±0.003 d
		*Rhopalosiphum padi* (Linnaeus)	−0.012±0.003 d
HIMENOPTERA	Aphelinidae	*Aphytis melinus* (De Bach)	0.025±0.002 d
LEPIDOPTERA	Gelechiidae	*Tuta absoluta* Meyrick	0.039±0.004 d
	Noctuidae	*Mythimna unipuncta* (Haworth)	0.059±0.004 d
		*Sesamia nonagrioides* (Lefevbre)	0.027±0.011 d
THYSANOPTERA	Thripidae	*Frankliniella occidentalis*(Pergande)	0.009±0.002 d

Absorbance values (±SE) at 492 nm obtained by indirect ELISA using the monoclonal recombinant antibody TJCc2 when tested against different total soluble protein extracts antigens (10 µg/ml) of related and less related arthropod species to *Ceratitis capitata*. 3% skimmed milk in PBS was used as negative control. Different letters indicates significant differences among means (Tukey’s test: *P*<0.05).

### SDS-PAGE and Western Blot

The specificity of both polyclonal and monoclonal sera was compared by western blot analysis using the soluble protein extracts from *C. capitata* and *D. melanogaster*. The soluble protein extracts were analyzed by one-dimensional SDS-PAGE on a 12.5% gel under reducing conditions. Protein bands were visualized by Coomassie blue staining. Electrophoretic transfer of proteins from gels to nitrocellulose membrane was performed using a Mini Trans-Blot Electrophoretic Transfer Cell (Bio-Rad). A pre-stained molecular weight marker (Bio-Rad) was used to estimate the loading and blotting efficiencies. Briefly, membranes were blocked overnight in blocking buffer I [PBS containing 4% skimmed milk], washed three times with TBS [10 mM Tris pH 7.6, 0.9% (w/v) NaCl, and 0.1% (v/v) Tween-20], incubated with a 1/15,000 dilution of rabbit polyclonal serum or 3×10^9^ pfu/ml of monoclonal recombinant antibody phage TJCc2 in blocking buffer II [PBS containing 2% skimmed milk] at room temperature for one hour, washed four times and then incubated with a horseradish peroxidase-conjugated (HRP)-conjugated anti-rabbit (1∶3,000 dilution in blocking buffer II; GE Healthcare) or HRP-conjugated anti-M13 (1∶1,000 dilution in blocking buffer II; GE Healthcare) at room temperature for one hour. After additional washing, blots were developed using the ECL Plus Western blotting detection reagents, as per manufacturer recommendations (GE Healthcare).

### Prey Detection in Predators

Live adults specimens of the wolf spider, *P. cribata* were collected from a citrus orchard located in the Valencia region (Spain) and placed individually into 150-ml plastic containers. Spiders were starved for seven days at 25°C and 16∶8 h (L:D) photoperiod. After starvation, each predator was offered one medfly adult from the IVIA 2000 colony. Spiders were allowed to feed on the medfly over a three-hour period, giving sufficient time for external digestion and ingestion phases following which, prey remains were removed and spiders (n = 8) frozen immediately. Seven days-starved spiders that were not fed were used as negative controls.

Total soluble protein extracts were obtained by homogenizing 1 mg of opisthosoma of each spider in 40 µl of PBS buffer at 4°C. The ability of selected phages to detect *C. capitata* antigens within the gut of the predator was tested by indirect ELISA. Selected phages were used at 4E+10 pfu. Each protein extract was tested in triplicate. Average absorbance readings obtained were compared to the negative control.

### Statistical Analysis

Serum reactivity of each antibody and antigen tested by indirect ELISA was analyzed using one-way ANOVA. Absorbance mean values obtained for the different antigen concentrations and serum dilutions were compared using Tukey’s tests (P<0.05). For the monoclonal antibody samples, absorbance values were log-transformed (log (x+1)) to correct for heterogeneity of variance.

## Results

### Rabbit Polyclonal Serum

**Figure 1:**
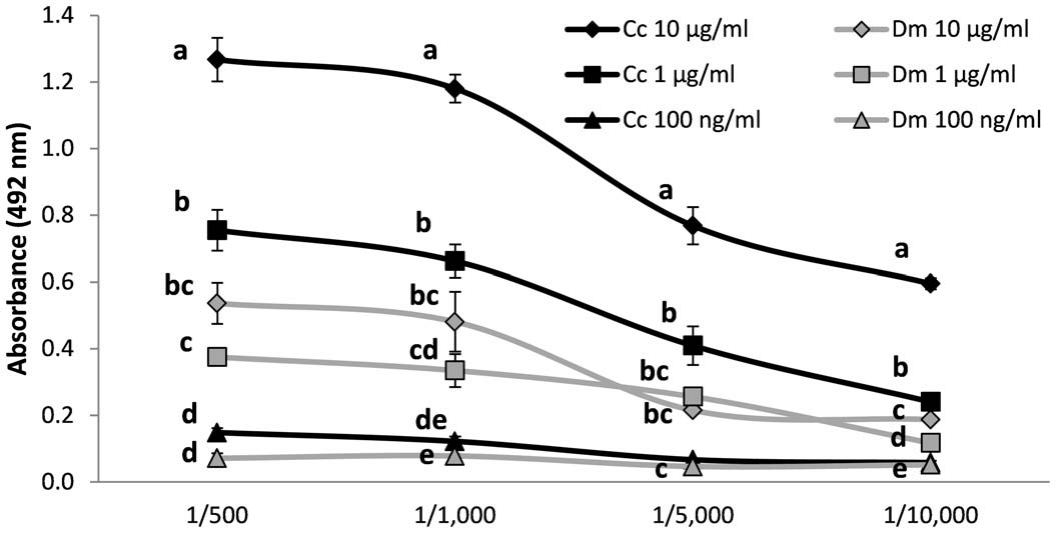
Indirect enzyme-linked immunosorbent assay (ELISA) response to rabbit polyclonal serum raised against total soluble protein extract of *C. capitata* (Cc) and *D. melanogaster* (Dm). Absorbance values (±SE) at 492 nm are displayed for different dilutions of the rabbit polyclonal serum and different protein concentrations (10 µg/ml, 1 µg/ml and 0.1 µg/ml) combinations of *C. capitata* and *D. melanogaster* total soluble protein extracts. For each serum dilution, different letters indicates significant differences among means (Tukey’s test: *P*<0.05).

Rabbit polyclonal serum raised against *C. capitata* extract was able to detect the presence of the target antigen at the lowest concentration tested (100 ng/ml) using indirect ELISA (Fig. 1). The serum was reactive to the antigen in dilutions up to 1/10,000. No reactivity was observed in the absence of antigen (MPBS). For each dilution, the serum reacted differently depending on antigen concentration (1/500: *F* = 88.00, *df* = 5, 20; *P*<0.0001; 1/1,000: *F* = 80.69, *df* = 5, 20; *P*<0.0001; 1/5,000: *F* = 51.96, *df* = 5, 20; *P* = 0.0012; 1/10,000: *F* = 670.1, *df* = 5, 11; *P*<0.0001). Serum dilutions of 1/5,000 and 1/10,000 were not able to detect *C. capitata* soluble protein at 0.1 µg/ml.

When the *C. capitata* and *D. melanogaster* soluble protein extracts were compared the highest reactivity was observed for *C. capitata* with absorbance values 2–3 folds higher for similar antigen concentrations. The absorbance values obtained for the 10 µg/ml *C. capitata* extract were significantly higher than for any *D. melanogaster* extract at all serum dilutions tested. However, 1 µg/ml *C. capitata* antigen gave significantly higher absorbance values only at the highest serum dilution (1/10,000) when compared to *D. melanogaster* antigen at all concentrations (Fig. 1).

### Monoclonal Recombinant Antibodies and Biopanning

**Figure 2:**
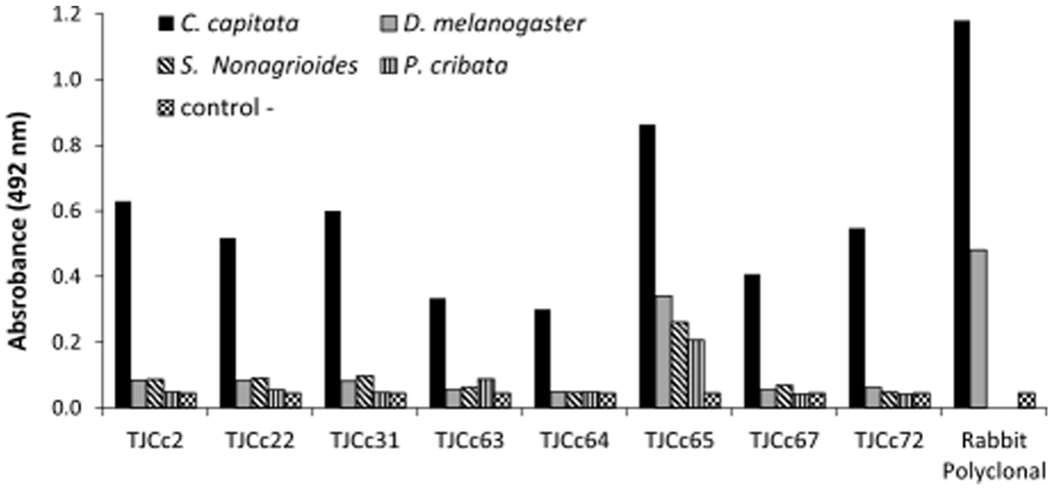
Indirect ELISA with selected monoclonal recombinant antibodies raised against total soluble protein extracts from *C. capitata*, *D. melanogaster*, *S. nonagrioides* and *P. cribata*. Absorbance values (±SE) at 492 nm are displayed for the selected monoclonal recombinant antibodies and total soluble protein extracts (10 µg/ml) combinations. Three percent skimmed milk in PBS was used as the negative control. The values obtained are compared with those determined for the rabbit polyclonal serum. Notice that the rabbit polyclonal serum was not tested against *S. nonagrioides* and *P. cribata* extracts.

After the third round of biopanning, 160 phage clones were isolated –80 clones for each phage library. In subsequent analysis of these clones, eight clones belonging to the Tomlison J library were selected based on their higher specificity and sensitivity to *C. capitata* antigens (TJCc2, TJCc22, TJCc31, TJCc63, TJcc64, TJCc65, TJCc67 and TJCc72). Clones selected from the Tomlison I library were discarded due to their low specificity and sensitivity values (data not shown). The eight clones selected from the J library displayed high specificity against *C. capitata* antigens when compared to antigens obtained from *D. melanogaster*, *P. cribata* and *Sesamia nonagroides* (Lefebvre) (Fig. 2).

### Sequencing, Alignment and Comparison of the V_H_ and V_κ_ Regions for the Selected Clones

**Figure 3:**
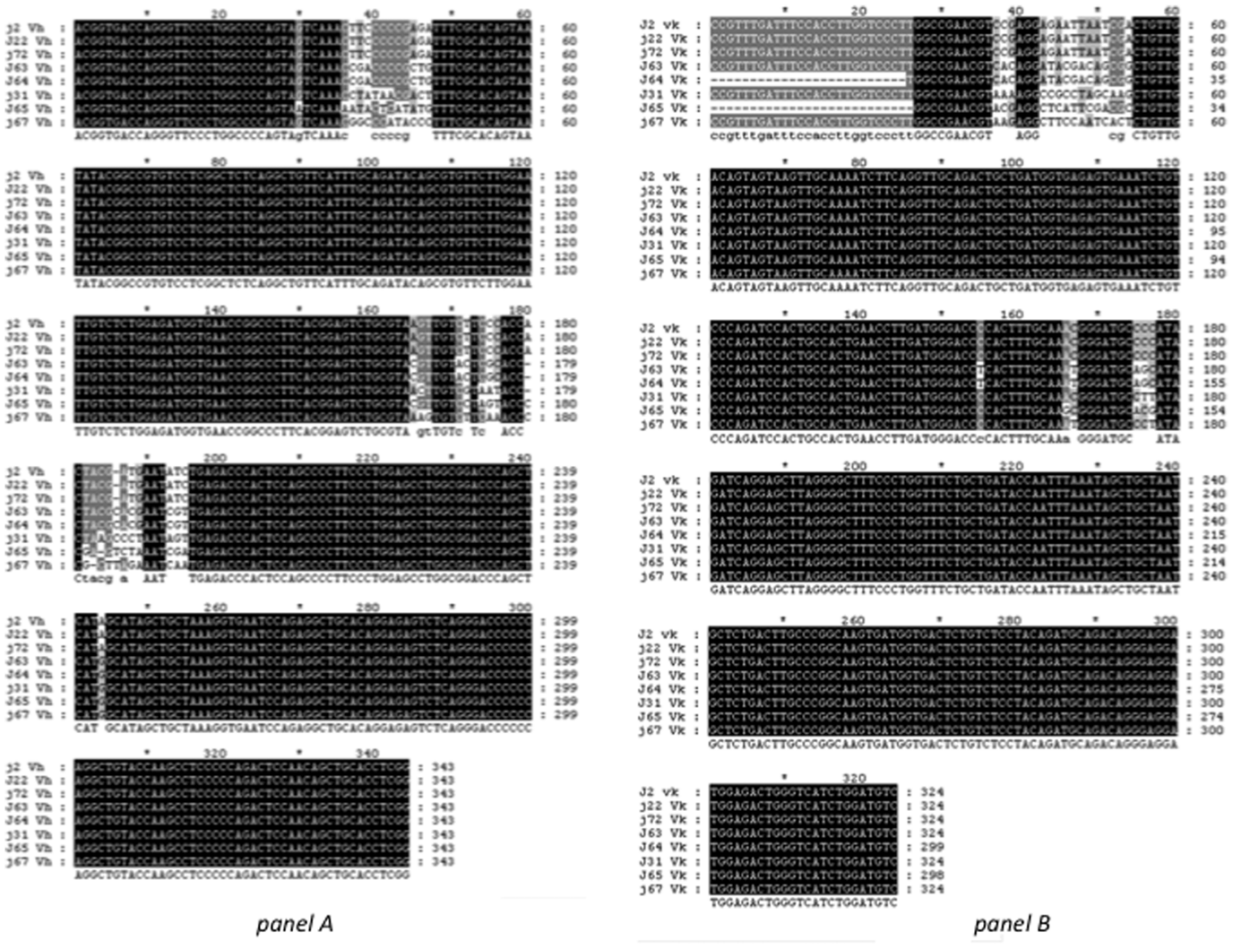
Sequence alignments of the VH and Vκ regions encoded by the selected clones. *Panel A*. Sequence alignment of the VH region. Black color indicates no differences among sequences (TJCc2: J2, TJCc22: J22, TJCc31: J31, TJCc63: J63, TJCc64: J64, TJCc65: J65, TJCc67: J67 and TJCc72: J72). Lighter colors indicate higher differences among the sequences for the nucleotides located in the same position. *Panel B*. Sequence alignment of the Vκ region. Black color indicates no differences among sequences (TJCc2: J2, TJCc22: J22, TJCc31: J31, TJCc63: J63, TJCc64: J64, TJCc65: J65, TJCc67: J67 and TJCc72: J72). Lighter colors indicate higher differences among the sequences for the nucleotides located in the same position. The first 25 and 26 nucleotides of the TJCc sequences were not sequenced.

In order to distinguish between novel antibodies, the V_H_ and V*_κ_* regions of the eight selected clones were sequenced and compared. It was found that the V_H_ and V*_κ_* regions of TJCc2, TJCc22 and TJCc72 were identical and TJCc63 and TJCc64 were also identical. Additionally, the TJCc31, TJCc65 and TJCc67 clones were unique (Fig. 3A and 3B). It is worth mentioning that the differences between clones appeared in both variable regions, and that this variability was due to a limited number of nucleotides. In the V_H_ fragment the variability appeared in three regions of approximately 10–15 nucleotides corresponding to the three hyper variable regions of the V_H_ fragment. The V*_κ_* fragment showed variability concentrated in two regions of 19 and 24 nucleotides, respectively. As expected, when the V_H_ and V*_κ_* sequences were compared in GenBank, the highest similarity was found with the HK102 precursor of the V-1 region sequence that codifies the *H. sapiens* immunoglobulin V*_κ_* region (92% identity, Gaps: 2/271, Expected 3e^−107^, for the V*_κ_* region), and with the LOC100291190 locus that codifies the *H. sapiens* immunoglobulin heavy chain variable region (89% identity, Gaps: 9/259, Expected 3e^−98^, for the V_H_ region).

### Specificity and Sensitivity Tests for the TJCc2 Clone

**Figure 4:**
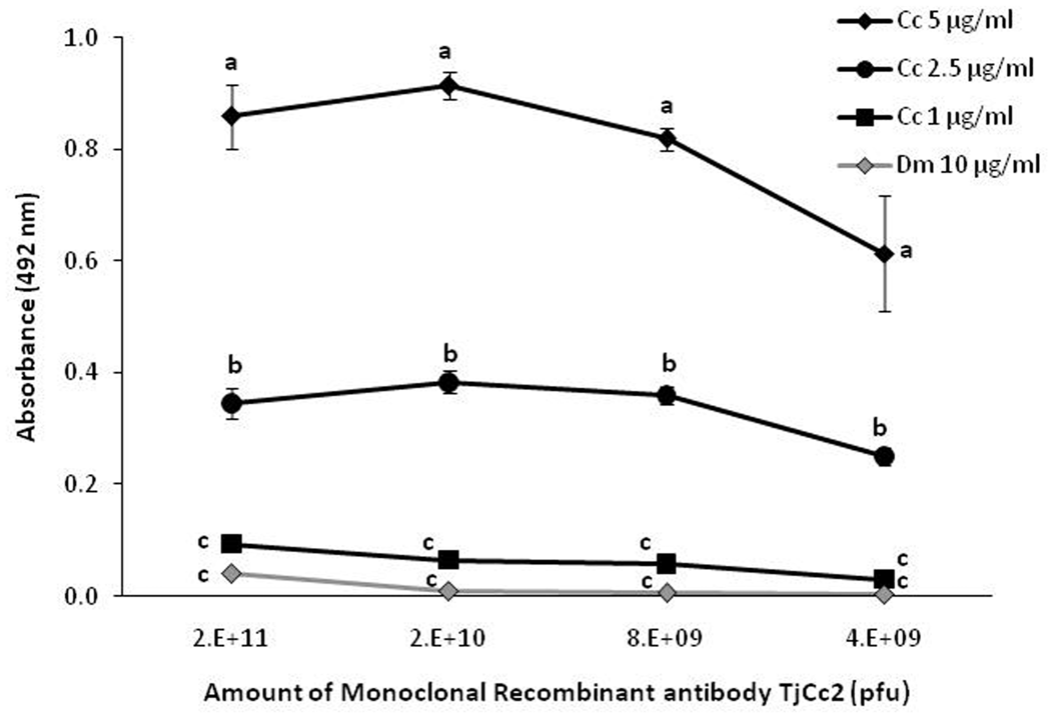
Indirect ELISA with the monoclonal recombinant antibody TJCc2. Absorbance values (±SE) at 492 mm obtained by indirect ELISA using different amounts of recombinant antibody TJCc2 against different protein concentrations (5 µg/ml, 2.5 µg/ml and 1 µg/ml) of *C. capitata* (Cc) and 10 µg/ml of *D. melanogaster* (Dm) total soluble protein extracts. Abreviations: pfu (plaque forming units) refers to the number of phage particles capable of forming plaques per ml. For each antibody dilution, different letters indicates significant differences among means (Tukeýs test: P<0.05).

Clone TJCc2 demonstrated highest specificity against *C. capitata* and was selected for further analyses. The ELISA absorbance values obtained against *C. capitata* antigens were significantly higher (P<0.05) than those of any related (Diptera) or less related arthropod species evaluated (Table 1). In the case of *P. cribata* and all the other unrelated species the absorbance displayed was statistically similar to that of the negative control (MPBS). The TJCc2 clone was less sensitive than the polyclonal antibody, detecting antigens at concentrations higher than 1 µg of protein/ml (Fig. 4). Nevertheless, the TJCc2 clone was still reactive to 2.5 µg of protein/ml of the *C. capitata* extract even using only 4×10^9^ pfu. It is noteworthy that the ELISA becomes saturated above 2×10^10^ pfu.

**Figure 5:**
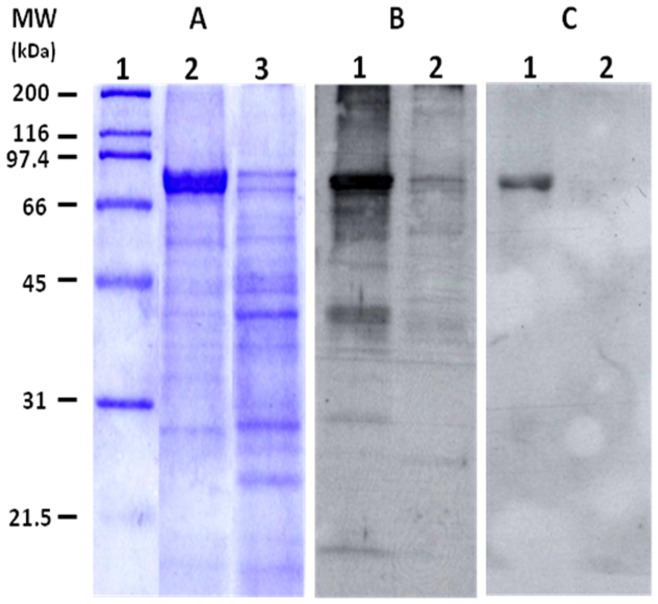
Western blot analysis. (A) Soluble protein extracts separated on 12.5% SDS-PAGE and stained with Coomassie blue. Lane 1, molecular weight markers (molecular weight (MW) is shown in kDa); lane 2, *C. capitata* extract (70 µg); lane 3, *D. melanogaster* extract (135 µg). (B) Western blot for total soluble protein extracts (4.5 µg each) using a rabbit polyclonal serum. Lane 1, *C. capitata*; lane 2, *D. melanogaster*. (C) Western blot for total soluble protein extracts (13.5 µg each) using the monoclonal recombinant antibody TJCc2. Lane 1, *C. capitata*; lane 2, *D. melanogaster*.

When the specificity of polyclonal and monoclonal antibodies was compared by western blot analyses (Fig. 5), the polyclonal antibody raised against total soluble protein extracts of *C. capitata* recognized a large number of proteins either from *C. capitata* or *D. melanogaster*, whereas the monoclonal antibody TJCc2 reacted specifically with the major protein of *C. capitata*.

### Prey Detection in Predators

Clone TJCc2 was able to detect *C. capitata* antigen in spiders that preyed on this species (mean ± SE absorbance at 492 nm = 0.150±0.028). On average, absorbance values were significantly higher than those obtained for the negative control (0.092±0.006) (*F* = 8.70, *df* = 1, 28; *P* = 0.007), though these values are far below the ones obtained with the *C. capitata* soluble protein extracts.

## Discussion

In this work we have for the first time selected and compared specific antibodies against the Mediterranean fruit fly, *C. capitata*, obtained from polyclonal serum raised in rabbit and from monoclonal antibodies generated from a commercial antibody phage display library. To our knowledge this is the first work that has selected and sequenced recombinant antibodies with potential use for post-mortem analysis studies.

The high specificity of monoclonal antibody TJCc2 was clearly demonstrated by western blot analyses (Fig. 5). The specificity of selected recombinant monoclonal antibodies was similar to or higher than that of classical monoclonal antibodies [15], [35]. In fact, the absorbance readings against *D. melanogaster* and other dipteran species closely related to *C. capitata*, were significantly lower than values obtained with *C. capitata* antigens. These differences were more evident with the predator *P. cribata* and with a wide range of other unrelated species that can be found in agroecosystems. In the case of the Mediterranean fruit fly predatory spider, *P. cribata* [29], [36] absorbance values did not differ from the negative controls. The sensitivity of all selected recombinant antibodies tested was lower than that of the polyclonal serum and of conventional antibodies found in the literature [16], [37], [38]. The low sensitivity of the selected recombinant antibodies may be due in part to screening a single antigen when compared with the multiple epitopes detected by polyclonal antibodies. In this sense, it is important to take into account that the polyclonal antibody was raised against a complete extract of the fly and not to a single antigenic determinant. In addition, lower sensitivity of the assay may be attributed to the use of the scFv fragments displayed on the phage capsule when compared to the soluble scFv fragments. The size of the antibody-phage complex (approximately 900 nm) is 120 times larger than a classical antibody (160 kD, approximately 7.5 nm) [39], [40]. This difference in size can decrease the likelihood of target antigen-antibody binding, thus reducing affinity and diminishing sensitivity. Interestingly, the selected monoclonal antibody (TJCc2) was able to detect the presence of the prey in spiders fed with *C. capitata.* However, it would be necessary to increase its sensitivity in order to be able to use this antibody in predation field studies. This problem could be overcome by using scFv purified soluble fragments produced in *E. coli* HB2151 transformed with the plasmids encoding the selected antibodies [41]. The soluble scFv fragments, with an approximate size of 26 KDa, are the smallest functional antibody domain currently used in immunology [42] and therefore, would be expected to increase affinity. Moreover, a major advantage assigned to recombinant antibodies is the possibility of constructing secondary libraries to improve characteristics such as affinity [23]. In fact, one of the greatest achievements of this technology has been the selection of recombinant antibodies with picomolar affinity [43], [44].

An important advantage of the technology described here is that the selection of specific recombinant antibodies against a target antigen can be done in less than two weeks. The advantages of using recombinant antibody technology include the reduced processing time, lower low costs, as well as the minimal level of expertise required when compared to the selection protocols for production of classic monoclonal antibodies. In the case of classical monoclonal antibody technology it can take at least 6 months to obtain the hybridoma lines [10] and expenditures can easily rise over US$20,000 per hybridoma [18]. Nevertheless, evaluations of the selected recombinant antibodies on the rates of antigen decay in the gut of the predator should be conducted to determine the detectability half-lives of these antibodies for the selected Mediterranean fruit fly predators [1].

The rapid advances in *in vitro* display technologies, with new libraries having clone variability several times higher than offered by any vertebrate or with libraries generated from specific antigens [16], [20], together with the capacity of sequencing and even synthesizing the selected antibodies, places this technique in a privilege position with respect to any classical method for immunodetection. The positive results obtained here for the selection of *C. capitata* specific recombinant monoclonal antibodies situate *in vitro* display technologies as a new alternative to classical monoclonal antibodies, or even molecular probes, in the post-mortem analysis studies of predator-prey systems. These findings have the potential to advance our understanding of the complex trophic interactions in conservation biological control programs, targeted to maintain or enhance predators that might be most efficient against this pest.
